# Discretion and local health policy implementation: street-level bureaucrats and integrative and complementary therapies in Santos’ local health units

**DOI:** 10.1017/S1463423622000172

**Published:** 2022-06-02

**Authors:** Ariel Macena, Vanessa Elias de Oliveira

**Affiliations:** Universidade Federal do ABC – Brazil, São Paulo, São Paulo 09606045, Brazil

**Keywords:** street-level bureaucracy, complementary therapies, public health policies, primary health care, local power

## Abstract

This research discusses contextual factors that influence the development of complementary and/or integrative therapies developed by local health units on street-level bureaucracy in Santos. Through a qualitative approach, the research verifies that street-level bureaucracy is free to suggest and implement the aforementioned therapies, even if they do not have formal support of the municipality; however, they need support from their immediate local supervisors so they can adjust and implement the practice’s routine, or the practice might not occur. Additionally, this text also presents guidelines in order to further develop the research.

## Introduction

Street-level bureaucracy is a topic widely explored in the literature regarding public policies owing to the vast number of professionals who play this type of role, but also due to its capacity to produce actions that go beyond what is pre-conceived by a specific public policy (Lipsky, 2010). There have been several studies regarding different subjects of street-level bureaucracies, such as their discretionary space and behavior (Winter, 2002; Evans, 2011; Hupe, 2013; Tummers and Bekkers, 2014; Akosa and Asare, 2017), decision-making processes and negotiation (Keiser, 2010; Loyens and Maesschalck, 2010; Johannessen, 2019), their point-of-view as professionals (Maynard-Moody and Musheno, 2000), accountability (Hupe and Hill, 2007; Lieberherr and Thomann, 2019), resilience to political influence (May and Winter, 2007; Stënsota, 2012) and innovation adoption (Arnold, 2014). As a professional who has the discretion to identify, apply and, if necessary, adapt public responses to the context brought by his/her client, the street-level bureaucrat becomes an important agent in understanding the materialization of public policy (Lipsky, 2010, Lotta, 2012). These actions performed by bureaucrats are taken under a guiding policy, which identifies and defines the best responses according to the need applied by the client (Lipsky, 2010). However, as the bureaucrat’s action occurs in the interaction with other individuals, the applied conduct is influenced by different contexts (such as professional ethics, peer influence, and a new client’s demand), which may result in actions not previously established (Dogaru, 2017). In this sense, the bureaucrat has the discretion to implement the action that he deems most resolutive based on the interpretation of his guiding policy, an action that may neither be easy to apply nor does it necessarily result in the path of least effort for him/her as a professional (Maynard-Moody and Musheno, 2000; Arnold, 2014; Dogaru, 2017). From the perspective of some scholars, such as Arnold (2021) and Cohen and Aviran (2021), there is an understanding that the bureaucrat may be a public agent of innovation, an *entrepreneur*, because his/her actions are a result of applied contextual analysis that seek to build the best outcome for that moment, considering the resources available and their needs as public professionals.

An applied debate on this issue is the realization of integrative and complementary health therapies – hereinafter called “practices” in this paper – by professionals from local health units in the Brazilian Public Health System – SUS (Aguiar, 2011; Brasil, 2015, 2018; Sousa and Tesser, 2017; Galvanese *et al.*, 2017). Integrative and Complementary Therapies are a set of practices recognized by the World Health Organization (WHO, 2013) that encompass traditional medicine, complementary medicine, herbal medicine, and other related practices in medical systems of greater or lesser complexity, which are locally applied as part of the health culture of a given population. They encompass beliefs, age-old knowledge, and tradition in an innovative way, aiming to meet the health needs of a population. When discussing complementary therapies, we consider different practices such as aromatherapy, homeopathy, herbal medicine, and meditation. According to Sousa and Tesser (2017), in Brazil, complementary therapies are primarily implemented in local-level units, which corresponds to 67% of the general practices developed in the country, stressing the importance that primary health care (PHC) plays in fostering the connection between community and healthcare professionals.

PHC is considered a robust model for fostering the health of a population, although its operationalization may be hard to achieve (Kluge *et al.* 2019). According to the World Health Organization (2018), “PHC is a whole-of-society approach to health that equitably aims to maximize the level and distribution of health and well-being by focusing on people’s needs and preferences (both as individuals and communities) as early as possible along the continuum from health promotion and disease prevention to treatment, rehabilitation and palliative care, and as close as feasible to people’s everyday environment.” As PHC develops according to a specific population’s needs, it is deeply dependent on Cultural, Economic, and Social bonding to foster its health promotion policy, thus making street-level bureaucrats’ unique characteristics a vital way to create and strengthen relations with and within the community. By recognizing the common features in their clients – in this paper’s context, the population related to a specific local-level unit –street-level bureaucrats are able to identify and provide the best practices to improve their clients’ health: as a population profile may change across different local-level units, so may the practices implemented. That characteristic highlights the particular importance of healthcare professionals who work at the frontline of local-level units: to promote an integrative approach of care within a given community by assimilating several populational traits and translating them into specific therapies and practices, bolstering PHC results, and reinforcing the connection between them.

Due to the fact that Brazil is a federalist country composed of three levels of constituent autonomous territorial units – the federal government, states, and municipalities – the existence of guiding policies on the federal level recommending a specific type of practice in a health area does not result in its enactment, *de facto*, at any particular level of government (Arretche, 2000; Sousa and Tesser, 2017). Likewise, a practice that is not regulated may occur as well, if the proper context is met. Therefore, not being regulated only means that a particular practice or policy does not receive formal resources through government budget for its implementation but ultimately it does not set itself as a type of obstruction of development of that practice or policy. This situation creates an optimal scenario to assess the discretionary actions of bureaucrats in primary care. In this context, the street-level bureaucrat may consider that providing complementary therapies is necessary to serve his/her target audience and, for this reason, carry them out, even if he/she does not have the necessary resources provided by the government. Hence, the bureaucrat articulates the necessary spaces and defrays the actions with his/her own resources, while, in the meantime, he/she seeks to present the empirical results of the actions in the hope that the government can institutionalize them.

Having presented these considerations, this paper discusses the factors influencing the implementation of recommended but unregulated practices by street-level bureaucrats in the Brazilian public health system. By recognizing the inherent discretion of these professionals, we discuss that street-level bureaucrats develop the aforementioned practices regardless of any financial incentives or policy standards to achieve their client’s well-being.

The present study was carried out with public health professionals in Santos, a medium-sized city with contrasting health regions, near São Paulo, the financial capital of Brazil. Santos is the fifth city with the best Human Development Index in Brazil (Instituto Brasileiro de Geografia e Estatística, 2013), standing out for the organization of its health system and the quality of its professionals. The municipality has a wide coverage of primary care in its territory, with 32 local health units, and an estimated population of 433 656 inhabitants (Instituto Brasileiro de Geografia e Estatística, 2013), approximately 70% of which are exclusively dependent on the public health system. Moreover, Santos does not have a structured local policy for the application of complementary therapies, creating a favorable context for the analysis discussed in the research.

The study identifies a set of factors that influence the actions of the street-level bureaucrat who implements complementary therapies, namely (1) professional hierarchy; (2) the profile and motivation of street-level bureaucrats; (3) peer influence; and (4) client and community influence. The analysis not only suggests that there is a predominance of some factors over others for the practice to take place, such as the professional hierarchy and motivation, but also states that other factors, such as peer influence, play an important role in the organization and dissemination of practices across the territory.

## Methodology

This study is an applied exploratory qualitative research (Yin, 2014). It seeks to answer the following question: which factors influence the implementation of non-regulated complementary therapies by street-level bureaucrats in local health units in the municipality of Santos and how do they do so?

The present research was structured in three distinct stages, namely (1) the realization of a survey to identify the local health units which, at the time of the study, carried out complementary therapies with their patients; (2) the application of semi-structured interviews with different health professionals aiming to identify the context of implementation of complementary therapies and the role of street-level bureaucracy in carrying out such actions; and (3) data analysis and development conclusions on the researched topic.

The first stage was based on a survey sent to the coordinators of the local health units in Santos to identify which of them performed complementary therapies based on the actions of their health professionals and to identify the types of therapies performed. The units were chosen among the results extracted from the survey, under certain criteria: (1) the choice of at least one unit in each health region of Santos; (2) the permission and availability to interview the professionals, during the data collection period; (3) the units needed to have at least two complementary therapies applied during the research period, one of them explicitly being “Let’s Move”; and (4) the duration of the practice.

The survey obtained 23 responses, of which 20 were considered valid (three of them were incomplete), encompassing approximately 2/3 of the local health units in the municipality (there are 32 units in total). Concerning complementary therapies, eight different practices carried out in the health units were mapped: (a) “Let’s Move!”, a group of physical education activities having the elderly as target audience; (b) integrative community therapy; (c) auricular therapy; (d) biodance; (e) circular dance; (f) music therapy; (g) reiki; and (h) phytotherapy. These responses were classified according to the research’s validation criteria, of which five units were selected for field interviews. The data collected are summarized in Table 1.

**Table 1.** Survey results

Health region	Local health unit	Does it have an ICT?	ICT	How long does each ICT exist? (in months)	Is the ICT practitioner at the unit nowadays?	Are interviews allowed?	EXTRA – Does it include “Let’s Move!”?
Northwest	USF Alemoa e Chico de Paula	Yes	Let’s Move!	+ 24	No	Yes	No
			Integrative Communitarian Therapy	0 a 6	No		
Northwest	USF São Manoel e Piratininga	Yes	Auricular therapy	—	No	No	—
Northwest	USF Castelo	Yes	Biodance	+ 24	Yes	Yes	Yes
			Circle Dance	+ 24	Yes		
			Let’s Move!	+ 24	Yes		
			Music therapy	+ 24	Yes		
Northwest	USF Bom Retiro	Yes	Circle Dance	+ 24	Yes	Yes	Yes
			Let’s Move!	+ 24	Yes		
			Integrative Communitarian Therapy	+ 24	Yes		
Northwest	USF Vila São Jorge e Caneleira	Yes	Reiki	6–12	Yes	Yes	No
			Let’s Move!	+ 24	Yes		
Northwest	SEUB Rádio Clube	Yes	Let’s Move!	0–6	Yes	No	No
Northwest	USF Areia Branca	No	Nonapplicable	Nonapplicable	Nonapplicable	Nonapplicable	No
Northwest	USF Piratininga	Yes	Let’s Move!	+ 24	Yes	Yes	No
Hills	SEUB Marapé	Yes	Let’s Move!	+ 24	Yes	Yes	No
Hills	USF Valongo	Yes	Integrative Communitarian Therapy	0–6	Yes	Yes	No
Hills	USF Monte Serrat	Yes	Let’s Move!	—	Yes	Yes	—
			Acupuncture	—	Yes		
Hills	USF Morro São Bento	Yes	Let’s Move!	+ 24	Yes	Yes	No
Coastal	SEUB José Menino/Pompéia	Yes	Let’s Move!	+ 24	Yes	No	No
Coastal	SEUB Campo Grande	Yes	Integrative Communitarian Therapy	+ 24	Yes	Yes	No
Coastal	SEUB Aparecida	Yes	Let’s Move!	+ 24	Yes	Yes	No
Coastal	SEUB Aparecida	Yes	Let’s Move!	—	Yes	Yes	—
Coastal	SEUB Gonzaga	Yes	Let’s Move!	+ 24	Yes	Yes	Yes
		Yes	Integrative Communitarian Therapy	+ 24	Yes	Yes	
Coastal	SEUB José Menino/Pompéia	Yes	Let’s Move!	+ 24	Yes	Yes	No
Coastal	SEUB Ponta da Praia	Yes	Let’s Move!	+ 24	Yes	Yes	Yes
		Yes	Integrative Communitarian Therapy	+ 24	Yes	Yes	
Coastal	SEUB Embaré	Yes	Let’s Move!	+ 24	Yes	Yes	No
Mainland	USF Caruara	Yes	Phytotherapy	+ 24	Yes	Yes	No
Mainland	SEUB Vila Mathias	Yes	Let’s Move!	+ 24	Yes	Yes	No

*Source:* Authors’ production.

The second stage of the research was conducted around the realization of semi-structured interviews together with the local health unit coordination and the professionals who were applicants of the aforementioned complementary therapies. Conversations with the bureaucrats of the municipal health department responsible for supervising the actions and policies of PHC were also included, verifying the role that municipal management has concerning the application of practices. Based on this, interviews were carried out with 13 different professionals, namely (a) five coordinators of a local health unit, (b) six street-level bureaucrats applying complementary therapies in local health units, and (c) two bureaucrats of the municipal health department responsible for observing the actions and policies of PHC. The description of the interviewees is summarized in Table 2.

**Table 2.** Interviewee profile

Interviews	Place of work	Gender	Educational level	Undergraduate degree (if applicable)	Professional position	First contact with an ICT
Interviewee 1	Municipality	Female	Bachelor	Odontology	Public Manager	Work
Interviewee 2	Hills	Male	Bachelor	Public Management	Local Health Unit Coordinator	University
Interviewee 3	Hills	Female	Technician	Community Health Agent	Community Health Agent	Work
Interviewee 4	Mainland	Female	Bachelor	Nursery	Local Health Unit Coordinator	University
Interviewee 5	Mainland	Female	Bachelor	Psychology	Community Health Agent	University
Interviewee 6	Hills	Female	Bachelor	Odontology	Local Health Unit Coordinator	Work
Interviewee 7	Hills	Female	Technician	Community Health Agent	Community Health Agent	Work
Interviewee 8	Northwest	Male	Bachelor	Not specified	Local Health Unit Coordinator	Work
Interviewee 9	Northwest	Female	Bachelor	Nursery	Nurse	University
Interviewee 10	Northwest	Female	Technician	Community Health Agent	Community Health Agent	Work
Interviewee 11	Municipality	Female	Bachelor	Odontology	Public Manager	Work
Interviewee 12	Coastal	Female	Bachelor	Odontology	Local Health Unit Coordinator	Work
Interviewee 13	Coastal	Female	Bachelor	Nutrition	Community Health Agent	Work

*Source:* Authors’ production.

The interview scripts were based on publications about street-level bureaucracy analysis, providing knowledge over categories and contextual factors that affect the actions of street-level bureaucrats, such as the studies of Anat Gofen *et al.* (2019) and Hill and Hupe (2019). However, the interview profile was designed to ask other questions and perceptions regarding the topic, expanding the analysis.

Furthermore, as the interviews were conducted, it was observed that other practices (such as aromatherapy and traditional Chinese medicine) also took place, even without the knowledge of the unit coordinator, and therefore are not mentioned in the present paper.

The third stage corresponds to the global analysis of the collected data, marked by Bardin’s (2011) and Minayo (2001) studies. The authors summarized and categorized the extracted data through an inductive process of coding, reflecting on the main questions raised in the interviews. The framework built identified four aspects that influence the implementation of non-regulated practices by street-level bureaucrats, namely:

1. professional hierarchy, discussing how the hierarchical structure within the public health department affects the implementation of non-regulated practices;
2. the profile of street-level bureaucrats, addressing motivational and professional competencies;
3. peer influence, such as the role played by teams in fostering the practices’ development; and
4. client influence, recognizing the different needs of the population living nearby local health units.

These categories were revisited several times by the authors in order to guarantee the recommended criteria of reliability and validity.

## Research analysis

The collected data were analyzed and classified according to the aforementioned categories, presenting the main types of observable effects on the implementation of complementary therapies by street-level bureaucrats in the local health units in Santos. In the following subsections, descriptions of each category will be presented, as well as their possible subcategories, presenting a summary of the interviews.

### Professional hierarchy

**Figure 1.**
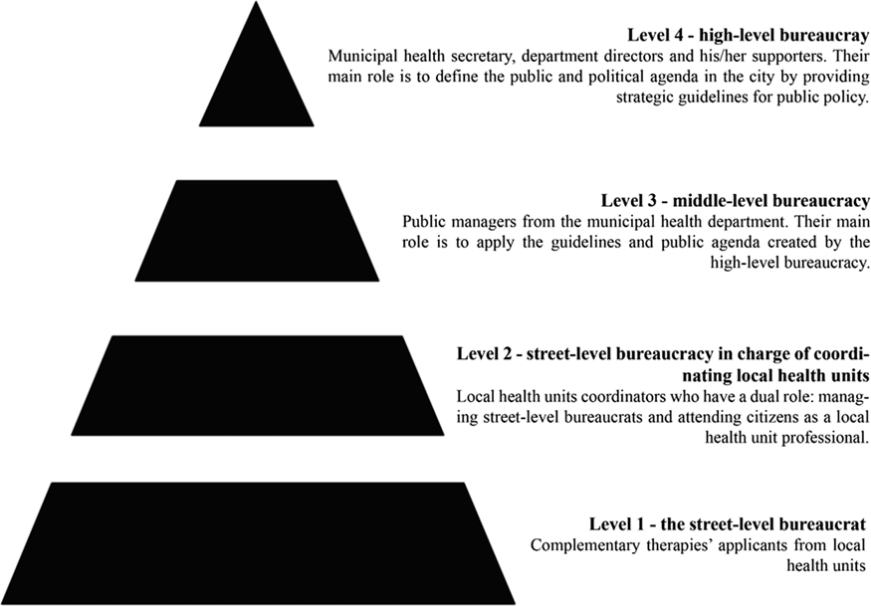
Bureaucratic levels of influence.Source: Authors’ production.

This category discusses the set of relationships and influences that different levels of bureaucracy at the public health system play in implementing complementary therapies at the local level. According to our analysis, it was observed that four bureaucratic levels are interrelated to enable the application of complementary therapies in local health units: (a) level 1, of street-level bureaucracy; (b) level 2, of the street-level bureaucracy in charge of coordinating local health units; (c) level 3, middle-level bureaucracy, composed of the technical areas of municipal health management; and (d) level 4, of high-level bureaucracy, composed of department directors and the municipal health secretary. The levels are organized according to Figure 1.

The main issues observed were separated into subcategories, presented below:

- **Level 1** – **the street-level bureaucrat**: according to Lipsky’s (2010) assumptions, he/she is responsible for bringing the practice to the unit, as he/she believes it fulfills the needs of the citizens. If the government does not formally support the therapy, he/she uses his/her own resources to maintain the action. He/she plays an essential role in originating the practice but does not have the means to maintain it for a long time.
- **Level 2** – **street-level bureaucracy in charge of coordinating local health units**: coordinators play a fundamental role in making practices feasible, as they adjust the unit’s service agenda and ensure that the street-level bureaucrats applying complementary therapies have available time to deploy the actions. The coordinator is not responsible for applying the practice, but he/she must ensure that the street-level bureaucrat has the ideal conditions for it to become viable.
- **Level 3** – **mid-level bureaucracy**: technical areas that accompany the activities developed in primary care act in two ways to support the application of complementary therapies: (a) not creating legal barriers for actions taken by street-level bureaucrats which are not formally displayed at municipal health policy; (b) seeking to create conditions for the practices implemented to be maintained, such as through technical administrative support for management and continuity of actions. The complementary therapy “Let’s Move!” is an example of practice which has informal support from mid-level bureaucracy. That is the reason why so many local health units mentioned having that specific practice in their routine.
- **Level 4** – **high-level bureaucracy**: this level is far from applying complementary practices, being remembered only when there is a need to provide financial resources for carrying out a particular policy. The discussion on the consolidation of this practice involves the need to regulate it, which can be undertaken at this level, as it is in charge of the public agenda of PHC.

### Profile and motivation of street-level bureaucrats

This category seeks to identify the role of personal characteristics in the design and implementation of complementary therapies in local health units. According to our analysis, it was possible to observe that there is a specific profile to the street-level bureaucrat professional who applies complementary therapies in the local health units in Santos: he/she (a) has increased recognition of the role of primary care; (b) is previously aware of the topic of complementary therapies; (c) recognizes complementary therapy as a pleasurable activity, not as work.

- **Heightened recognition of the role of primary care**: street-level bureaucrats recognize that primary care is essential for the prevention and promotion of health; hence, in addition to the traditional service recommended, they understand that other actions which may contribute to the health of their clients are also essential and must be implemented, including complementary therapies.
- **Sensitization to the topic of complementary therapies:** professionals applying complementary therapies have already had previous contact with the practices, either because some member of their immediate family (siblings, spouse, relatives and/or children) performed them or because they have taken courses in therapies, or by themselves applying them in their daily lives; however, there is always an empirical recognition of the benefits that the practice produces.
- **Recognition of the activity related to complementary therapies as something pleasurable, not work:** street-level bureaucrats understand these actions as something unrelated to their daily work, enabling a pleasant experience for everyone. Hereupon, the action produces a rewarding feeling for the professionals, reinforced by the citizens’ gratitude.

However, all this motivation has a limit: if bureaucrats encounter adverse situations for a long time, such as lack of resources or political and/or administrative support, they stop acting. This is aligned with the proposition made by Lipsky (2010) about how street-level bureaucrats cope with everyday issues.

### Peer influence

This category seeks to identify the health unit team’s role in the performance of complementary therapies by one of its professionals. According to our analysis, it is possible to notice that peers influence the dissemination of the practice in the community.

- **Disseminating practices in the community**: the team has an essential role in supporting the dissemination of practices among clients of local health units. As it is not regulated, the practices depend on a “word of mouth” campaign to be maintained and expanded to different citizens. Thus, communication and referral to participating in complementary therapies by each professional support the practice as a whole. For this, however, the professional who acts as a referee must also believe that the practice he/she is indicating is effective, or otherwise this incentive will not occur.

Professionals in health units who do not value complementary practices do not disseminate actions among their patients, but neither do they prevent other professionals from carrying them out.

### Client influence

This category analyzes the relationship of the bureaucrat with the client, considering different pressure criteria that the community exerts over the procedures and activities sustained by the local health unit’s team. Here, three subcategories were built: (a) social demand for carrying out specific kinds of practices; (b) the recognition of the needs endured by vulnerable groups; (c) practices as a way of strengthening community bonds.

- **Social demand for carrying out practices**: street-level bureaucrats who carry out complementary therapies recognize the role of pressure played by the population. When clients hear news of practices and actions that other health units carry out, they pressure the professionals of their reference units so that the same practice is also available to them, playing an active role in producing such practices.
- **Recognition of the needs endured by vulnerable groups**: street-level bureaucrats understand that the needs of different groups, especially those with high vulnerability, such as the elderly, pregnant women, and hypertensive patients, are a motivating factor for them to continue performing complementary therapies, even in a context of scarce resources. This factor is also a trigger for new practices, corroborating the theoretical discussion on the production of new actions by street-level bureaucrats (Arnold, 2014, 2021; Cohen and Aviran, 2021).
- **Practices as a way of strengthening community bonds**: street-level bureaucrats see benefits not only for citizens but also for themselves, especially for their work. Professionals report that strengthening the bond through complementary therapies is reflected in greater participation of citizens in the regular activities of prevention and health promotion and even in the allowance of the local health unit’s team into their homes during routine visits.

## Key findings

This research noted that a large number of contextual factors influence the performance of complementary therapies by street-level bureaucrats, with direct impacts on PHC. In this direction, if, on one hand, the theoretical understanding of the theme of public policy implementation is broad, on the other, there is still scope for exploration and integration of the theme with other fields of research, such as public health (Oliveira, 2016).

A relevant issue is a discretionary space that exists in the structure of the Brazilian public health system. Local health units within the same city may have diverse and divergent services, even within the same health region. That corresponds to the understanding of PHC as intended by WHO (2018), relating it to the provision of a set of practices coherent with the local context. As street-level bureaucrats act as a bridge between the local health services and the population, they are free to negotiate and implement practices addressing health community challenges (Johannessen, 2019). Professionals carry out these actions despite formal regulation of such practices, requiring only support of the local health unit’s coordinator. As argued by Thomann *et al.* (2018), discretion is a quasi-essential condition for street-level bureaucrats to redefine and implement policies adherent to the clients’ needs, affecting their willingness to develop new practices.

As easy as they emerge, such practices also disappear. Hence the importance of social mechanisms, such as social pressure through neighborhood associations and councils, to engage health professionals: not only may the practices become perennial at a specific unit, but they also may become part of the city’s public health policy, reinforcing the link with PHC. This case reaffirms the policy entrepreneur role proposed by Arnold (2021) and Cohen and Aviran (2021), stressing that a way to cope with clients’ demands may be related to innovative practices implemented at the front lines of government.

Another aspect to highlight is the understanding that street-level bureaucrats have of their commitment as health professionals and the actions that can improve the quality of their client’s life. Thus, complementary therapies, despite occurring as part of the bureaucrat’s work, are not seen by him/her as work in itself, but as an action similar to voluntary work, whose final reward is the client’s well-being (Filho and Borges, 2014; Souza *et al*., 2015). This action is manifested in the bureaucrats’ speeches when they mention carrying out daily practices on themselves or family members, reinforcing the identification with their benefits and bonds that they elicit. This argument, however, does not align with the idea proposed by Arnold (2021) that street-level policy entrepreneurs act to maintain the *status quo*: there is evidence that recognition of clients’ needs by frontline healthcare professionals may increase the pressure over which practices are and are not developed within a specific local health unit; it also does not comply with the understanding that street-level bureaucrats’ actions are developed as a way to facilitate its work, as intended originally by Lipsky (2010) and followed by other authors (Winter, 2002; Keiser, 2010; Loyens and Maesschalck, 2010). On the other hand, the argument does state that street-level bureaucrats can create new practices if the proper context is met (Arnold, 2014; Dogaru, 2017), identifying them as entrepreneurs.

The role that peers play in carrying out complementary therapies is also crucial, as they are vectors for disseminating practices among citizens. By getting to know different individuals’ profiles in their procedures and attendances, they support the street-level bureaucrat who applies the practice by issuing direct invitations to participate in the actions promoted by the latter. In her article, Arnold (2017) debates the importance networks play in street-level policy entrepreneurship, relating them to a vital resource to innovate. The findings of this study corroborate this argument. However, it is important to notice that this support is not always genuine, as the referring professional must recognize the results that complementary therapies bring to the client; as scientific evidence of complementary therapies results is scarce, other professionals may not cooperate if their views of such practices are not aligned with that of the practitioner.

Finally, a significant factor is the role played by different bureaucratic levels (Gofen *et al.* 2019). Understanding the kind of action that each type of bureaucracy takes (if it is creating or providing technical support, building an agenda, etc.) is essential for the government to enable complementary therapies to grow and expand in scale. Bureaucracies operate to a limited extent in their areas, but correct integration of their resources can offer scalability of actions and a thorough improvement in the quality of public health services.

## Conclusion

This research elicited a somewhat explored interdisciplinary discussion, which is the development of complementary therapies by street-level bureaucrats, based on an approach that observes the contextual factors that influence the implementation of such practices. This paper contributes to understanding how non-regulated PHC practices may develop within local health units, helping to identify the challenges and opportunities faced by teams in providing care to a given population. It also broadens the current understanding of what motivates street-level bureaucrats to act as street-level entrepreneurs, presented by Arnold (2021), by showing that it is not only the maintenance of current conditions that mobilize them but also an accurate recognition of its clients’ needs, resembling these actions to volunteering.

However, there is a need for further exploration of this subject by speaking with professionals who do not value complementary therapies; this could create a set of information that may be of interest to public managers who may need to implement new practices in environments whose health professionals have not yet been sensitized for such an issue. Moreover, it is viable to explore cost-benefit aspects of practices relative to public welfare, identifying arguments that may be of interest to the highest levels of the bureaucracy, creating a “window of opportunity” for the implementation of a complementary therapy’s public policy. In addition to the latter, it may also be possible to foster the role of prevention, one of the central elements of PHC.

The research also supports the debate around implementation of innovative actions in the scope of PHC for health systems worldwide. Understanding the factors that favor the realization of new practices, from their emergence to their maintenance and replication, creates new possibilities for health systems and their relationship with health promotion in their populations.

It is also necessary to recognize the importance of the discussion on interdisciplinary studies between public policies and health. Using analytical methods from one disciplinary field to address recurring themes in other disciplinary fields is a way to deepen the debate on themes that are already known, but which can benefit from new tools and models for their understanding. Health is an essential aspect for governments, and the insertion of this point of view in the analysis of different public policies contributes to the evolution and efficiency of the systems as a whole.

Another point that should be noted is the limitations of this paper. This research was conducted with few interviewees due to the COVID-19 pandemic breakout in Brazil, which occurred during the period in which field research interviews were carried out. In addition to the small number of possible participants due to the research criteria, this is a relevant blindspot in this paper. Further studies should be developed with more participants in order to improve the arguments presented here.

Finally, this paper has shown that complementary therapies occur despite the existing regulations, as there is ample discretionary space on the part of street-level bureaucrats working in local health units for their realization. In order to implement complementary practices, street-level bureaucrats need motivation and support from the local health unit’s coordinator. Other factors mentioned in the paper, such as peer support and middle-level bureaucracy, play an important role not in the creation of the practice but in its maintenance and/or scalability. Henceforth, recognizing and valuing the performance of such street-level bureaucrats are essential, as they support the health system to continue innovating in its care and in strengthening relations with the population.
